# Dendritic cell-based immunotherapy in non-small cell lung cancer: a comprehensive critical review

**DOI:** 10.3389/fimmu.2024.1376704

**Published:** 2024-09-06

**Authors:** Jamile Barboza de Oliveira, Saulo Brito Silva, Igor Lima Fernandes, Sabrina Setembre Batah, Andrea Jazel Rodriguez Herrera, Andrea de Cássia Vernier Antunes Cetlin, Alexandre Todorovic Fabro

**Affiliations:** ^1^ Department of Pathology and Legal Medicine, Ribeirão Preto Medical School, University of São Paulo, São Paulo, Brazil; ^2^ Department of Medical Imaging, Hematology and Clinical Oncology, Ribeirão Preto Medical School, University of São Paulo, São Paulo, Brazil; ^3^ Neuropathology and Molecular Biology Division, Bacchi Laboratory, Botucatu, Brazil; ^4^ Pulmonary Division, Internal Medicine Department, Ribeirão Preto Medical School, University of São Paulo, São Paulo, Brazil

**Keywords:** lung cancer, dendritic cells, immune system, tumor microenvironment, checkpoint inhibitors, immunotherapy, cancer vaccine

## Abstract

Despite treatment advances through immunotherapies, including anti-PD-1/PD-L1 therapies, the overall prognosis of non-small cell lung cancer (NSCLC) patients remains poor, underscoring the need for novel approaches that offer long-term clinical benefit. This review examined the literature on the subject over the past 20 years to provide an update on the evolving landscape of dendritic cell-based immunotherapy to treat NSCLC, highlighting the crucial role of dendritic cells (DCs) in immune response initiation and regulation. These cells encompass heterogeneous subsets like cDC1s, cDC2s, and pDCs, capable of shaping antigen presentation and influencing T cell activation through the balance between the Th1, Th2, and Th17 profiles and the activation of regulatory T lymphocytes (Treg). The intricate interaction between DC subsets and the high density of intratumoral mature DCs shapes tumor-specific immune responses and impacts therapeutic outcomes. DC-based immunotherapy shows promise in overcoming immune resistance in NSCLC treatment. This article review provides an update on key clinical trial results, forming the basis for future studies to characterize the role of different types of DCs *in situ* and in combination with different therapies, including DC vaccines.

## 1 Introduction

Non-small cell lung cancer (NSCLC) places a significant emotional, physical, and financial burden on individuals and their families, healthcare systems, and society in general in terms of lost productivity (1). Because nearly 75% of patients do not receive a diagnosis until stage III or IV, and many recur or progress, long-term outcomes are unfavorable, with only about 19% of patients achieving 5-year overall survival (OS) (2).

Immunotherapies that block the immune checkpoint programmed cell death protein 1 (PD-1) receptor and its ligand (PD-L1) have become the standard backbone therapy in the treatment of NSCLC lacking actionable genetic alterations, improving patients’ outcomes in a range of clinical scenarios (3–5). However, the quest for novel approaches that can enhance the clinical benefits of immunotherapy for the majority of NSCLC patients has driven the evaluation of targeting other co-inhibitory immune blockades (LAG-3, TIGIT) or inhibitory soluble molecules (adenosine) (6).

Despite therapeutic advances, tumor cells inevitably acquire immune resistance through various mechanisms, with non-explicit targets (such as non-druggable receptors or molecules) standing out as significant barriers to treatment due to their ability to establish resistance to known immunotherapy approaches. Inappropriate T-cell priming by antigen-presenting cells (APCs) and/or inadequate tumor-cell recognition via the major histocompatibility complex (MHC) may indeed serve as the Achilles’ heel in establishing robust and long-lasting tumor-immunological responses, posing challenges to overcoming immune resistance (7).

Among APCs from the mononuclear phagocytic system (including DCs, monocytes, and macrophages), DCs are the most adept at promoting the activation of naïve T cells. They are characterized by their ability to produce cytokines in the presence of antigens and pathogen-associated molecular patterns (PAMPs) or damage-associated molecular patterns (DAMPs). These APCs influence the intensity of the inflammatory response, especially in areas of constant contact between the environment and the host, such as the lung (8–10).

DCs, or professional APCs, are hematopoietic-derived stem cells that infiltrate pathological tissues, searching for antigens to trigger effector lymphocyte responses (7). These cells – so called due to the many cytoplasm projections resembling a “tree”, from the Greek prefix *déntro* – operate on two fronts, both in the cellular and tumor immune response, through initiation, regulation, and in defense against infections. DCs reside in different tissues and use specialized surface receptors to capture antigens, such as endocytosis receptors, phagocytosis receptors, and C-type lectin receptors (7). After encountering antigens and being exposed to inflammatory stimuli, these cells migrate to the lymph nodes and spleen, where they activate non-sensitized T cells and undergo the maturation process. During this process, they lose their capacity to capture and process antigens and increase the expression of MHC-I and MHC-II molecules and co-stimulatory receptors (CD40, CD80, CD86) (7, 11, 12). DCs help to regulate the immune response mediated by T lymphocytes, determining the balance between the Th1, Th2, and Th17 profiles and the activation of Treg, depending on the nature of the antigens, cell-cell interactions defined by key receptors, and the environmental cytokine milieu within which they are exposed (13).

Every DC originates from a common adult hematopoietic stem cell precursor that differentiates under the influence of the receptor tyrosine kinase Flt3 (CD135) (14) and a distinct set of transcription factors (15, 16). These cells can be found in steady-state conditions or as a consequence of inflammation or microbial stimuli. Steady-state DCs are subclassified as conventional DCs, responsible for orchestrating immune responses to cancer, comprising two major subsets: type-1 cDCs (cDC1s) and type-2 cDCs (cDC2s) (17, 18). The expression of basic leucine zipper transcription factor (BATF3) and IRF8 over IRF4 defines cDC1 lineage, whereas IRF4 expression is crucial for cDC2. The cDC1 cells are devoted to activating CD8+ T cells, whereas cDC2 cells are ontogenically and functionally diverse, focusing on presenting exogenous antigens to CD4+ T cells to initiate helper T cell differentiation. In tumor-specific immune responses, cDC1s are key players, presenting tumor antigens to CD8+ T cells through MHC-I cross-presentation and driving Th1 differentiation among CD4+ T cells (19, 20). Although cDC2s may promote Th2/Th17 responses, their role is less understood (18).

## 2 Advances in the characterization of pulmonary DCs

The human lung has two unique microenvironments for DC subsets: the airways themselves and the interstitial spaces. However, the exact identities of these subsets are only beginning to be understood based on surface markers and transcriptional profiles. The low frequencies of DC subsets in lung tissues and the difficulty in isolating them have impaired their functional characterization, as a large amount of source tissue is required to obtain enough cells for substantial functional assays. Another factor contributing to this low frequency is the dynamic exclusion of functional DCs by lung cancers from the tumor microenvironment, aiding in malignant progression (21).

Although human DC subsets have traditionally been recognized through a limited selection of markers, such as CD1c^+^, CD141^+^, and CD14^+^ (22), this simplistic phenotypical representation fails to capture the complexity of DC identity in various physiological processes and tumoral microenvironments (TME) (23). Recent advancements in flow cytometry methods have enabled a more detailed analysis of DC subsets, redefining subset-specific phenotypes with greater precision (24). Moreover, integrating surface markers data with transcriptome analysis, mainly through single-cell transcriptome analysis, has allowed for the comprehensive characterization of different DC subsets throughout dynamic processes (25). The single-cell RNA sequencing (scRNAseq) approach offers a valuable framework for exploring how DC transcriptome undergoes reprogramming within the tumor setting, providing a foundation for designing new therapies targeting DCs (26).

Recent scRNAseq experiments using lung tissue have identified a new subset of DCs, termed DC3, which exists on a phenotypic spectrum between monocyte-like and cDC2-like cells (27). This subset arises not from a common hematopoietic stem cell precursor but from granulocyte-macrophage colony-stimulating factor (GM-CSF)-dependent inflammatory precursor cells and is capable of activating the expansion of resident memory T cells (T_RM_) (28). Targeting T_RM_ may offer a promising strategy to restore T cell exhaustion and enhance the efficacy of immunotherapies (29, 30).

Additionally, single-cell analysis in NSCLC has revealed that pDCs in primary tumor tissues and metastatic lymph nodes exhibit immunosuppressive phenotypes that may influence anti-tumoral responses (31). These pDCs are characterized by the upregulation of leukocyte immunoglobulin-like receptor (LILR) family genes and granzyme B (*GZMB*) production, while showing a loss of the B7 family immune-regulatory ligands (CD80, CD86), which are essential for T cell activation (31).

## 3 Dendritic cells and anti-tumoral responses

The ability of DCs to sense and interpret danger signals, traffic to draining lymph nodes, and effectively present antigens is fundamental to developing anti-tumoral immune responses. These processes are closely linked to the maturation of DCs. The initial activation of DCs through Toll-like receptor (TLR) signaling is crucial for releasing proinflammatory cytokines, mainly IL-12, and upregulating MHC Class I and II molecules – hallmarks of DC maturation and activation. Indeed, the administration of exogen TLR ligands can activate DCs within TME, thereby supporting Th1-driven immune responses, CD8+ T cell and NK cell activation, increased IFN production, and enhanced migration of antigen-presenting cells to lymph nodes in response to the chemokine receptor ligand 7 (CCR7) (8, 32). Efficient TLR ligand-therapies, such as imiquimod (a ligand for TLR7 and TLR8) and CpG oligodeoxynucleotides (ligand for TLR9), have been demonstrated to activate immune responses across various cancers (33–35).

Within the TME, cDC1s play a crucial role in anti-tumor immunity by cross‐presenting tumor antigens to CD8+ T cells via MHC class I molecules (19, 36). Additionally, cDC1s sustain anti-tumor responses by activating CD4+ T cells through the chemokines CXCL9 and CXCL10 (20). Studies have shown that intratumoral DCs expressing the key cDC1 markers, such as CD141+ and BATF3, correlate with improved survival and response to immune checkpoint inhibitors (ICIs) (37, 38). Furthermore, intratumoral DCs exhibiting high expression of lysosomal‐associated membrane protein 3 (LAMP3) and CCR7 are associated with long-term survival in patients with NSCLC and melanoma, respectively (37, 39).

After encountering T cells for antigen presentation, another crucial step in activating T-cell towards anti-tumoral responses involves the engagement of the CD40 receptor on DCs with CD40L on activated T cells (24). This interaction upregulates co-stimulatory molecules, such as CD80 (B7.1) and CD86 (B7.2), on the surface of DCs, further stimulating IL-12 production and activating effector CD8+ T cells with non-exhausted phenotypes and high IFN-γ secretion capacity (24). CD40 signaling also prolongs DC survival by reducing tumor-induced apoptosis (40) and helps counteract the immunosuppressive effects of IL-10 produced by tumors (41).

## 4 Dendritic cells and immune tolerance

Although DCs are critical in modulating immune responses, their dysfunction can significantly contribute to immune evasion and tumor progression (25). This dysfunction can arise from their poor migration ability into tumors, resulting in low intratumoral DC counts and the expulsion of functional DCs from lung tumor lesions. This expulsion leads to a failure to induce molecular expression necessary for effective immune responses, thereby promoting immunosuppressive environments. Additionally, interactions with various cells and molecules within the TME can further modulate DC function, impacting overall antitumor immunity (26, 42). Identifying these mechanisms is crucial for designing more effective antitumoral immunotherapies, either by enhancing the activity of functional DCs or by blocking the immunosuppression induced by tumor cells. Furthermore, understanding the immune crosstalk and cooperative interactions between DCs and various TME components, such as tumor cells, lymphocytes, innate immune cells, and stroma cells, is essential to unlock the full potential of DC-based treatments (11, 29).

NSCLC cells can orchestrate DCs to secrete immunosuppressive cytokines that stimulate the differentiation and expansion of Tregs, a subset of immune cells known for their potent suppressive activity. Schneider et al. (43) demonstrated that NSCLC recruited immunosuppressive DCs characterized by the expression of B7-H3, a programmed death ligand (PD-L) family member. These cells secreted elevated levels of IL-10 and TGF-β, and reduced levels of IL-12, which critically contributed to the predominance of Tregs within primary lung tumors. In another study, Dumitriu et al. (44) demonstrated that lung carcinoma-induced DCs diminished the manifestation of IL-12, CD86, and HLA-DR, thereby contributing to the generation of Tregs (CD4^+^CD25^+^Foxp3^+^) within the TME, which suppressed the proliferation of T CD8+ lymphocytes. Of note, Schneider et al. (43) showed that antibodies against B7-H3 repair the T cell stimulatory capacity of DCs, while the study by Perrot et al. (32) demonstrated that, in NSCLC, the subset of DCs characterized by CD11c^high^ expression could reverse from a nonactivated/immature phenotype (impaired IL-12 and IFN-α expression) to a mature phenotype (expressing MHC, IL-12, and IFN-α) after stimulation by the TLR ligands TLR4 and TLR8. Additionally, TLR ligands were shown to upregulate the chemokine receptor CCR7, which plays a crucial role in antigen transport by DCs to draining lymph nodes in order to effectively prime T CD8+ cells through MHC-I cross-priming (32, 45).

## 5 Dendritic cell vaccine therapy

The basic principles of immunotherapies for cancer can be categorized into active and passive methods. Active cellular immunotherapy targets tumor-associated antigens (TAAs) to stimulate the immune system against cancer (46). On the other hand, passive cellular immunotherapy enhances the activation phase of immune cells by administering cytokines, antibodies, or immune cells to patients, potentially resulting in autoimmunity and adverse toxicity due to its lack of specificity in the immune response (11).

Vaccination of DCs represents a promising approach within active cellular immunotherapy. Two main techniques for obtaining DC vaccines are *ex vivo* differentiation of DCs and direct targeting of antigens to DCs *in vivo.* The *ex vivo* approach uses leukapheresis on the patient to collect CD14+ monocytes or CD34+ hematopoietic stem and progenitor cells (HSPCs). These cells are then differentiated into immature DCs in the presence of granulocyte-macrophage colony-stimulating factor (GM-CSF) and interleukin-4 (IL-4) while being pulsed with TAAs or tumor cell lysates, together with stimulation in a maturation cocktail comprising TNF-α, IL-1β, IL-6, and PGE_2_ (47). Once maturated and loaded with TAAs, these DCs can be administered to the patient via subcutaneous, intravenous, intradermal, intranodular, or intralymphatic routes. However, the effectiveness of these mature DCs may be compromised when inserted into an immunosuppressive *in vivo* environment (48).

**Figure 1:**
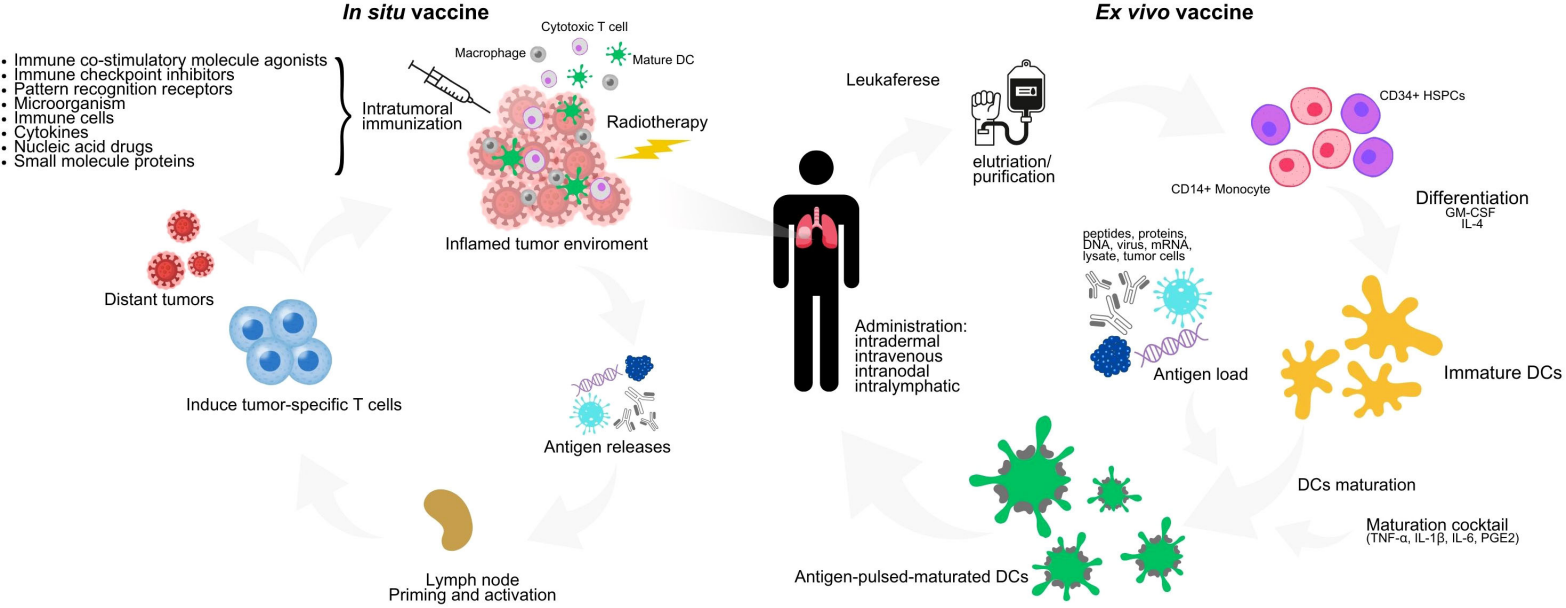
*Ex vivo* and *in vivo* DC vaccine scheme. In the *ex vivo* technique, peripheral blood mononuclear cells such as CD14+ monocytes or CD34+ HSPCs are collected from patients by leukapheresis, differentiated into immature DCs in the presence of GM-CSF and IL-4, and then matured in a maturation cocktail consisting of TNF-α, IL-1β, IL-6 and PGE_2_, while pulsed with autologous tumor cell lysates or TAAs. Mature DCs loaded with antigen are reinfused into the patient. The *in vivo* technique is based mainly on radiotherapy and intratumoral injection. For the preparation of intratumoral immunization, there are two divisions: 1) by the mechanism of action which can be divided into pattern recognition receptor (PRR) agonists, ICIs, DCI inducers, tumor antigens, cytokines and others and 2) by the type of preparation which can be divided into pathogens (bacteria, viruses), cells, nucleic acids, proteins (antibodies, small molecule proteins). Radiotherapy induces immunogenic cell death, which is an important step in establishing *in situ* vaccines. DC, dendritic cells; GM-CSF, granulocytemacrophage colony-stimulating factor; IL-4, interleukin 4; TNF-α, tumor necrosis factor α; PGE_2_, prostaglandin E2; HSPCs, hematopoietic stem and progenitor cells; IL-1B, interleukin 1-beta; IL-6, interleukin 6.

Another strategy to address the limitations of *ex vivo* manipulation involves directly targeting antigens to endogenous DCs *in vivo*. This approach leverages the tumor itself as a vaccine to stimulate both local and systemic anti-tumor immune response, and can be achieved through radiotherapy and intratumoral immunization (49) (**Figure 1**). The effectiveness of this strategy depends on the quality of the antibody-conjugated antigens and the adjuvant used. These adjuvants can include microorganisms (such as viruses or bacteria), synthetic compounds that mimic infectious agents (like pattern recognition receptor agonists), immunomodulatory monoclonal antibodies, cytokines, and chimeric proteins (50). This method aims to reduce the risk of systemic toxicities and achieve higher local concentrations of bioactive agents by ensuring local absorption (51).

The primary focus in selecting tumor antigens for vaccine therapy should be on tumor-specific immunogenic antigens that would not be expressed in that cell under standard conditions (52). Various sources of antigens, including tumor cell lysates, exosomes, and TAAs, have been used to produce an efficacious DC vaccine (53). Current clinical trials are exploring using DCs as a means of reactivating the immune system by restoring antigen presentation failure or T-cell co-stimulatory signals and inducing anti-tumor immunity, either by itself or in association with ICIs (2, 54, 55).

Despite the potential of DC vaccines, the lack of standardized methods for obtaining, maturing, loading antigens, and administering DCs represents a significant challenge (47). Future advances lie in combining DC surface targets and selected adjuvants to achieve the desired immunological results, which will require a great deal of research in the coming years (56).

## 6 Dendritic cell therapy in NSCLC

Initial studies suggest that DC vaccine is efficacious in improving the survival of NSCLC patients. However, despite its well-established immunogenicity, DC-based immunotherapy still offers poor response rates (57).

Takahashi et al. (58) conducted a multicenter clinical trial in Japan, administering intradermal DC vaccines every two weeks to 260 patients with advanced NSCLC. Patients were injected five or more times at sites close to the axillary and/or inguinal lymph nodes, and 0,1 ml of the vaccine was administered on the forearm to assess response to erythema after 24 to 48 hours. The DC vaccines were pulsed with Wilms’ tumor gene 1 (WT1) and/or mucin 1 (MUC1) synthetic peptides, used as tumor antigens in the six participating institutions. The study found that an erythematous reaction at the injection site was a favorable prognostic factor for mean survival time (MST) from the first vaccination. Specifically, an erythematous reaction with a diameter of ≥30 mm was strongly correlated with OS (≥30 vs. < 30 mm: MST 20.4 vs. 8.8 months, P < 0.001). During an era when advanced NSCLC patients were treated solely with chemotherapy, the authors concluded that DC vaccines provided meaningful clinical benefit for a subset of patients, especially those with adenocarcinoma.

Fong et al. (59) performed a study based on serum levels of carcinoembryonic antigen (CEA), an intercellular membrane adhesion glycoprotein overexpressed in various tumors. In this study, patients with metastatic or relapsing NSCLC with elevated or dysregulated CEA levels were identified and received prior dosing with Flt3 ligand, a hematopoietic growth factor known to expand DCs *in vivo*, before undergoing peripheral blood leukapheresis. DCs were then collected, loaded with a non-peptide derived from a CEA peptide specific to the human leukocyte antigen (HLA)-A0201, and infused intravenously with progressive doses of antigen-exposed DCs. The clinical responses showed a significant relationship with the expansion of CD8+ T cells and promising results, which may justify that several studies have chosen CEA as the standard antigen for active immunotherapy with DCs (59, 60). For studies involving DC vaccination targeting CEA, side effects are minimal, in contrast to the occasional serious side effects seen with checkpoint inhibitors.

Chunlei Ge. et al. (53) conducted an open-label, dose-escalation phase I clinical trial involving 15 patients with resected stage I to IIIA NSCLC. They evaluated a modified autologous DC vaccine pulsed with survivin, a member of the apoptosis-inhibiting protein family, and MUC1, silenced with suppressor of cytokine signaling 1 (SOCS1) and immunostimulated with flagellin, a specific ligand for TLR5. Patients were treated with the vaccine at 1x10^6^, 1x10^7^ up to 8x10^×7^ on days 7, 14, and 21, with good tolerability even at the highest doses. There was a reduction in the number of CD3 + CD4 + CD25 + Foxp3+ Treg and an increase in TNF-α and IL-6, indicating an improvement in the differentiation of DC and their antigen presentation characteristics. Furthermore, 11 out of 15 patients included had no recurrence in the long-term follow-up.

These new DC-based vaccination strategies are promising therapies for patients with NSCLC, but efficacy and safety still need to be evaluated in larger populations and prospective studies.

## 7 Exploring combination therapies

DC vaccination has been explored with other cancer treatments to overcome immunotherapy resistance and enhance therapeutic outcomes. These combination strategies aim to boost the immunogenicity of tumors and improve patient responses. The integration of various immunotherapeutic approaches represents a promising frontier in cancer therapy, but it also presents the challenge of effectively combining and optimizing these treatments to achieve maximum efficacy (**Table 1**).

**Table 1:** Use of DCs as adjuvant therapy isolated or in combination with the standard of care in NSCLC. Searching on clinicaltrials.gov (accessed on July 9th, 2024) using the keywords “dendritic cells”, “Non-Small Cell Lung Cancer”, “immunotherapy”, “DC-based vaccine”, “DC-based vaccine against cancer”, “DC-based vaccine and immune checkpoint”, “DC-based vaccine and immune checkpoint blockers” and “DC-based vaccine and CIK”.

Clinical trials testing DC vaccine-based immunotherapy in NSCLC patients						
Strategy	Phase	Status	Classification tumor	Life Expectancy/Performance status	Associated treatment	Reference
Anti-PD-1/​PD-L1 Treatment +/​- UV1 Vaccination	II	Recruiting	IIIB/IIIC, IV	n.i./ECOG 0-2	Sagramostim	NCT05344209
AST-VAC2 (Allogeneic Dendritic Cell Vaccine)	I	Completed	advanced	>12 wk./ECOG 0-2	n.a.	NCT03371485
Autologous DC adenovirus CCL21 intratumoral vaccine	I	Terminated	IIIB, IV, or recurrent	n.i./ECOG 0-2	n.a.	NCT01574222
Autologous DC adenovirus CCL21 vaccine	I	Terminated	IIIB, IV, or recurrent	n.i./ECOG 0-2	n.a.	NCT01574222
Autologous DC adenovirus CCL21 vaccine	I	Active, not recruiting	IV	n.i./ECOG 0-1	Pembrolizumab	NCT03546361
Autologous DC adenovirus CCL21 vaccine	I	Completed	IIIB, IV, or recurrent	n.i./ECOG 0-2	n.a.	NCT00601094
Autologous DC adenovirus CCL21 vaccine	I	Active, not recruiting	IV	n.i./ECOG 0-1	Pembrolizumab	NCT03546361
Autologous DC vaccine	II	Completed	I, II, III	n.i./ECOG 0-1	n.a.	NCT00103116
Autologous DC vaccine loaded with personalized peptides (PEP-DC vaccine)	Ib	Recruiting	IIIA, IVB	n.i./ECOG 0-1	Cyclophosphamide	NCT05195619
Autologous dendritic cells pulsed with antigen peptides ID	I/II	Unknown	progression	> 6 mo./ECOG 0-1	Nivolumab IV	NCT04199559
Combination of γδ T cells/DC-CIK	I/II	Completed	II,III,IV	> 3 mo.	Cryosurgery, γδ Tcells/DC-CIK	NCT02425748
DC vaccine subcutaneous administration	I	Recruiting	postoperative	> 3 mo./ECOG 0-1	n.a.	NCT04147078
DCVAC/LuCa	I/II	Completed	IV	n.i./ECOG 0-1	Chemotherapy, immune enhancers	NCT02470468
DCVAC/LuCa added to Chemotherapy	II	Unknown	IV	n.i./ECOG 0-1	Pemetrexed, carboplatin	NCT02669719
Intratumorally-administered Ilixadencel in Combination With Checkpoint Inhibitor	I	Terminated	n.i.	n.i./ECOG 0-1	Pembrolizumab	NCT03735290
mRNA Vaccine	I/II	Completed	metastatic	n.i./ECOG 0-2	Durvalumab, tremelimumab	NCT03164772
PDC*lung01 vaccine, associated or not with anti-PD-1	I/II	Active, not recruiting	IIa/Iib, IIIa, IV	n.i./ECOG 0-1	Keytruda, Alimta	NCT03970746
Recombinant Human rEGF-P64K/Montanide Vaccine	II/III	Terminated	IIIb/IV	n.i./ECOG 0-2	n.a.	NCT00516685
Therapeutic Cancer Vaccine, PDC*lung01, Associated or Not With Anti-PD-1	I/II	Active, not recruiting	IIa/IIb/IIIa, IV	n.i./ECOG 0-1	Keytruda, Alimta	NCT03970746
Vaccination With Autologous DC pulsed with Allogeneic Tumor Lysate (MelCancerVac)	II	Completed	advanced/metastatic	n.i./n.i.	Cox-2 inhibitor of celecoxib	NCT00442754
Vaccination With Tumor Antigen-loaded Dendritic Cell-derived Exosomes	II	Completed	advanced unresectable	n.i./n.i.	Metronomic cyclophosphamide (mCTX)	NCT01159288
Vaccine Therapy, Tretinoin, and Cyclophosphamide	II	Completed	IV	n.i./ECOG 0-1	Cyclophosphamide, ATRA	NCT00601796

ECOG, Eastern Cooperative Oncologic Group; n.i., not informed; n.a., not applicable; wk., week; mo., month.

### 7.1 Dendritic cell therapy and chemo/radiotherapy

The clinical effectiveness of traditional chemotherapy in treating tumors may not solely depend on its direct effects against tumor cells, but restoring immunosurveillance can also play a role. Some chemotherapeutic agents induce tumor cell immunogenicity and alter the properties of tumor-infiltrating lymphocytes, thereby supporting immune infiltration (34). Conventional chemotherapeutic drugs that induce immunogenic cell death include cyclophosphamide, temozolomide, and gemcitabine. Furthermore, some chemotherapy drugs are known to induce immunogenic cell death and temporary lymphoablation, reduce immune suppressor cells, and increase anti-tumor T-cell response (61).

Several studies have considered the immunostimulatory potential of chemotherapy. Hu et al. (62) evaluated the effectiveness and safety of co-culturing autologous tumor lysates with pemetrexed plus DCs as second-line therapy for 27 patients with advanced lung adenocarcinoma. Although this study did not use a control group with pemetrexed in monotherapy, it did show promising clinical effects with such a combination, as demonstrated by 4.5 months in median PFS and 10.5 months in median OS (62, 63).

Zhong et al. (56) conducted a phase II, prospective, open-label, single-arm study to evaluate the safety and efficacy of DCVAC/LuCa, which consists of autologous DC pulsed *ex vivo* with killed NSCLC H522 cell lines, combined with carboplatin/pemetrexed as first-line therapy for 61 patients with advanced non-squamous NSCLC without oncogenic drivers. Patients without progression after two cycles of chemotherapy began receiving subcutaneous DCVAC/LuCa for up to 15 doses. No significant adverse effects related to leukapheresis or vaccination were observed. Survival rates were 52.57% at two years, and progression-free survival was 8.0 months, indicating promising efficacy.

Zemanova et al. (64) conducted a Phase I/II, multicenter, randomized, open-label, three-arm clinical trial to evaluate DCVAC/LuCa combinations for stage IV NSCLC. Patients were divided into three groups: group A (45 patients): DCVAC/LuCa and chemotherapy (carboplatin and paclitaxel); group B (29 patients): DCVAC/LuCa, chemotherapy, pegylated interferon-α2b and hydroxychloroquine (this group was later discontinued); or group C (38 patients): chemotherapy alone. DCVAC/LuCa was administered subcutaneously at 3-6 weeks intervals, with a maximum of 15 doses. The combination of DCVAC/LuCa with carboplatin and paclitaxel showed a median OS of 15.5 months, compared to 11.8 months in the chemotherapy arm. These results demonstrate the efficacy and tolerability of the combined therapy.

**Figure 2:**
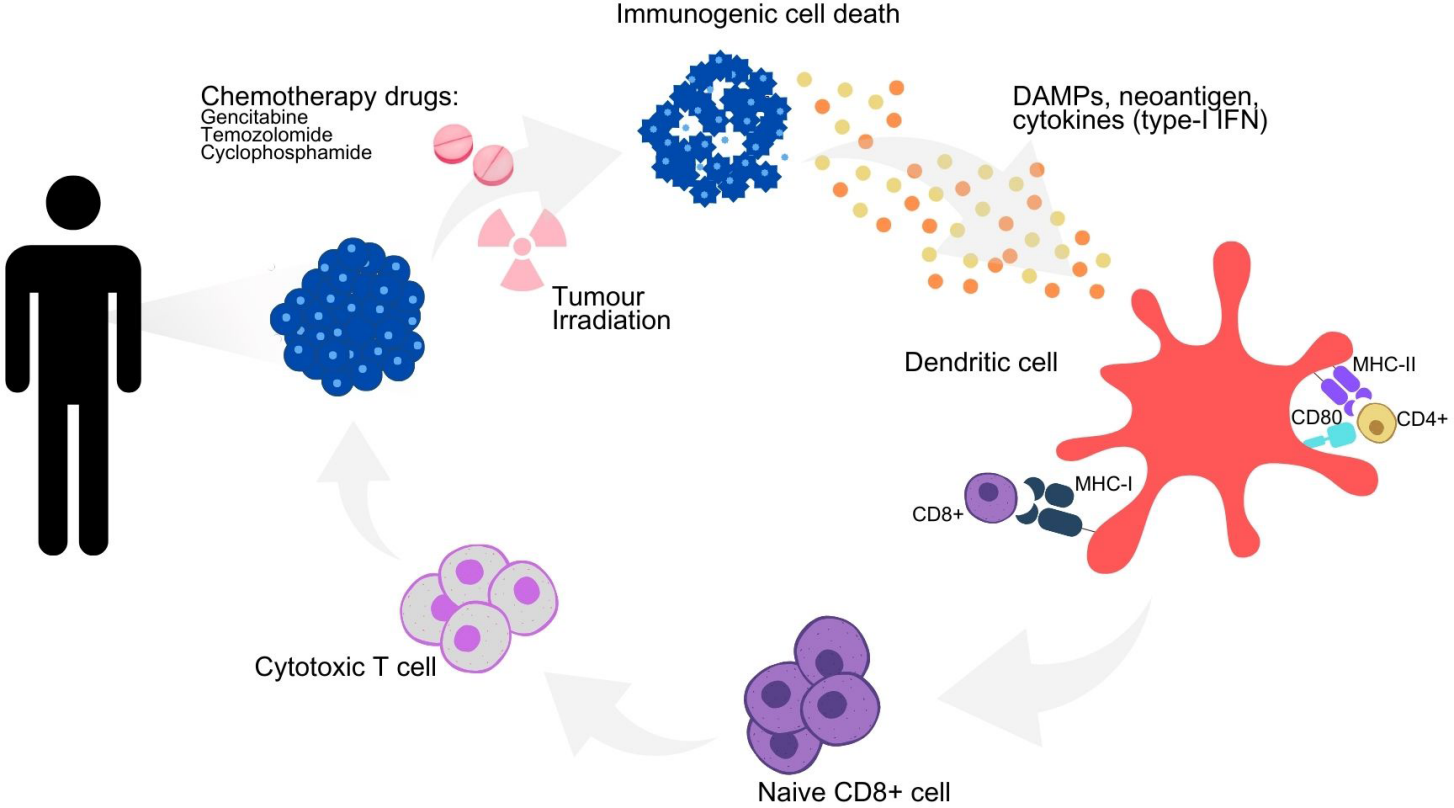
Immunomodulatory effects of chemoradiotherapy on immune activation and immunogenic cell death. Schematic representation of the immunogenic cell death cycle, with the release of DAMPs, neoantigens and cytokines from a dying tumor cell, leading to the maturation of a DC and the activation of CD4+ and CD8+ T cells, which in turn trigger the death of the remaining living tumor cells. Combination strategies to maximize the therapeutic efficacy of DC-based immunotherapy and their underlying mechanisms of action. DAMPs, danger-associated molecular patterns; IFN type-I, type-I interferons; MHC I, major histocompatibility complex I; MHC II, major histocompatibility complex II.

Radiotherapy can enhance systemic anti-tumor immune responses through various immunomodulatory mechanisms (65). It acts as a cytotoxic agent that damages DNA, triggers immunogenic cell death, and releases DAMPs and tumor-derived antigens into the tumor microenvironment (**Figure 2**). This process initiates systemic anti-tumor immune responses by activating DCs, which are then transferred to lymph nodes, thereby inducing anti-tumor immune responses at sites distant from the irradiated site (11). Radiation exposure also leads to the release of type I IFN from cancer and immune cells and activates the complement system, which further activates DCs and T-cells (32). With several studies published, the synergy between radiotherapy and immunotherapeutic agents in NSCLC has garnered considerable interest in developing immunotherapies, particularly ICIs. Although this approach shows promise, combining radiotherapy with DC-based immunotherapy awaits further validation through prospective studies.

### 7.2 Dendritic cell therapy and immune checkpoint inhibitors

The potential synergistic effect of combining DC-based vaccines and ICIs offers significant hope for NSCLC patients. Therapies that stimulate DCs are considered ideal complements to ICIs, analogous to “pressing the gas pedal” and “losing the brakes” simultaneously (66, 67).

Recent studies provide valuable insights concerning the synergistic effect of DC-based therapies and ICIs. One highlighted the role of Batf3-dependent DCs in cross-priming tumor antigens to CTLs, which up-regulates PD-1 in immune cells. Consequently, the expansion and activation of Batf3-dependent DCs and anti-ICIs offer promising combined antitumor therapy (38). Other studies have also shown that therapies focused on stimulating DC activity, such as Flt3L and TLR3 agonists, can restore the efficacy of anti-PD1 therapy (68).

In a preclinical experiment, Garris et al. (69) demonstrated the possibility of improving anti-tumor immunity through cross-talk between T cells and DCs using non-canonical NF-kB agonism alongside PD-1 therapy. Employing single-cell RNA sequencing (scRNAseq), the researchers discovered that a subset of DCs must infiltrate the tumor and produce interleukin-12 (IL-12) to enable an effective anti-tumor response. Although these DCs did not bind to anti-PD-1, they could recognize interferon γ (IFN-γ) secreted by adjacent T cells to produce IL-12. Thus, complete activation of anti-tumor T cells requires both IFN-γ and IL-12, which are essential immunological agents for tissue-specific destruction (69).

Laheurte et al. (70) investigated the interaction between the intratumoral signature of pDCs and the efficacy of ICIs. Compared to healthy donors, NSCLC patients exhibited a low-activated pDC phenotype. The study found that patients with high levels of pDC, NSCLC in stages I to III, and without metastatic disease, had better OS than patients with low pDC levels, with a mean OS of 30.4 versus 20.7 months (P = 0.013). It was also concluded that the best clinical outcomes were observed in patients with high levels of pDC in the tumor microenvironment who had undergone treatment with anti-PD-L1.

Dalil Hannani et al. (67) developed a cell line based on allogeneic plasmacytoid dendritic cells (PDC*line) from the blood of a patient with plasmacytoid DC leukemia to demonstrate a cytotoxic T-cell response against tumors in the presence Pembrolizumab. The PDC*line cells were loaded with peptides that induce robust T-cell responses (NY-ESO-1, CAMEL, MAGE-A2, MAGE-A3, and MAGE-A9). It was observed that MAGE-A3 specific T cells increased threefold in the presence of ICI. This study, involving 26 patients, highlighted advancements in combining ICI with PDC*line cells, demonstrating an improvement in the breadth and scope of the specific T-cell response in NSCLC.

Lee et al. (71) conducted a clinical study to evaluate the safety, efficacy, and anti-tumor immune response in patients with stage IIIB/IV NSCLC who received intratumoral vaccination with autologous DCs transduced with an adenoviral vector expressing CCL21 gene (Ad-CCL21-DC). The study found no evidence of virus dissemination or unintended antibody formation. It demonstrated a marked infiltration of CD8+ T cells into the tumor, indicating a robust local immune response. Additionally, there was a notable enhancement in systemic immune responses against tumor-specific antigens and an increase in PD-L1 expression on tumor cells, suggesting a potential for ICIs efficacy. This clinical trial highlighted that combining DC-based intratumoral vaccination with ICIs could synergistically boost immune responses in NSCLC patients.

These recent researches aim to provide predictive biomarkers for the efficacy of DC-based therapies combined with ICIs, as cross-priming against tumor neoantigens appears to be a key determinant of the efficacy of these treatments. Enhanced vaccine effectiveness may be achieved through immune screening, targeting cross-primed DC populations, or differentiating these cells from precursors in culture (38).

### 7.3 Dendritic cell therapy and cytokine-induced killer cells

First identified in the 1990s, Cytokine-Induced Killer (CIK) cells are a mixture of T-cell and NK-like phenotypes (72). It is now understood that these cells undergo significant proliferation and possess the ability to suppress tumors. They are considered safe, are not restricted to a single MHC molecule, and are effective anti-tumor agents, making them a promising candidate for cancer immunotherapy (73). The CIK cells consist of a combination of T cells (CD3 + CD56-), NKT cells (CD3 + CD56+), and NK cells (CD3 - CD56+). Among these, the CD3+ CD56+ phenotype has the highest amount of granzymes, making them responsible for most of the antitumor activity and cytotoxicity (72, 74).

Research has demonstrated that DCs, as professional APCs, can compensate for the lack of tumor antigen specificity of CIK cells. This combination of DC-CIK cells has shown potent lytic and anti-tumor activities, effectively preventing tumor cell growth (74).

Zhu et al. (75) conducted a study involving 63 patients diagnosed with stage IIIB NSCLC, randomly assigned to either a study or control group, to evaluate the effectiveness of DC-CIK combined with concurrent radiochemotherapy. The study group of 30 patients was given DC-CIK therapy alongside conformal radiotherapy and docetaxel-cisplatin chemotherapy, while the control group of 33 patients received the same therapy but without DC-CIK. The study group showed a significantly higher Karnofsky performance score (KPS) (83.3% vs. 54.5%; p = 0.014), improved T-cell subsets, and a higher 12-month survival rate compared to the control group, indicating notable benefits of the combination therapy in enhancing patient quality of life and survival.

Yang et al. (76) enrolled 122 advanced NSCLC patients. Of these, 61 received chemotherapy alone, while another 61 received chemotherapy along with DC-CIK cells. The treatment group displayed a significantly higher OS rate than the control group (57.2 vs. 37.3). The researchers evaluated the immunologic reactions in 10 patients pre- and post-treatment but found no change in T lymphocytes with CD3+ and CD3+ CD8+ phenotypes. Nevertheless, therapy led to an expansion of CD3+ CD56+ cells, and no severe side effects like high fever, chills, or anemia were reported, suggesting that DC-CIK therapy is superior to standard chemotherapy.

Additionally, combining DC vaccination with immunotherapy and CIK cells has been shown to reduce the rate of Tregs and cancer recurrence among NSCLC patients, indicating that this combined approach effectively enhances anti-tumor immunity and improves patient outcomes (42).

## 8 Conclusion and future perspectives

DC-based chemoimmunotherapy has established itself as a significant treatment model in cancer immunotherapy over recent decades (77), and current immunotherapy approaches focus mainly on increasing the antigen-presenting activity of DCs (72). However, as established in this review, published studies have several common limitations: the low number of patients subjected to the study, the absence of a control group in most cases, lack of a standardized method (including the differentiation of the maturation state of DCs after vaccination), the different use of concomitant immunostimulants, and the different routes and frequencies of DC infusion (78). Optimizing each of these issues could improve the clinical performance of DC-based treatments.

A significant advancement is the evidence that DC vaccination, as a cellular and self-activated immunotherapy, has proven effective in treating some solid tumors (56). However, despite the significant advances, there are major challenges to overcome. A notable obstacle is the lack of reliable biomarkers for pre-selecting patients to guide the application of DC-based vaccines, such as tumor mutational load or PD-L1 positivity for ICBs (79).

The future of cancer immunotherapy will likely be based on a dual approach: the first focuses on interrupting the immunosuppression triggered by the tumor, while the second aims to stimulate anti-tumor immunity. CD-based immunotherapy has already been integrated with other cancer treatments, such as chemotherapy, radiotherapy, and ICIs. This integration is crucial in treating NSCLC (80) and is already part of several ongoing clinical trials. However, phase III clinical trials completed in the field are still limited and, to date, have failed to show a significant advantage gained through DC-based vaccines (79).

Although there is still much work to be done to achieve the ideal universal or personalized immunotherapy based on DCs, it is hoped that recent advances and upcoming clinical trials will encourage the therapeutic implementation of DC-based vaccines in the near future.
